# Prevalence and factors associated with pre-diabetes and diabetes mellitus in Kenya: results from a national survey

**DOI:** 10.1186/s12889-018-6053-x

**Published:** 2018-11-07

**Authors:** Shukri F Mohamed, Martin Mwangi, Martin K Mutua, Joseph Kibachio, Abubakar Hussein, Zachary Ndegwa, Scholastica Owondo, Gershim Asiki, Catherine Kyobutungi

**Affiliations:** 1 Health and Systems for Health Unit, African Population and Health Research Center (APHRC), Nairobi, Kenya; 2 Division of Health Sciences, Warwick Medical School, University of Warwick, Coventry, UK; 3 Field Epidemiology and Laboratory Training Programme, Ministry of Health, Nairobi, Kenya; 4 NCD unit, Ministry of Health, Nairobi, Kenya; 5 The Institute of Global Health, Faculty of Medicine, University of Geneva (UNIGE), Geneva, Switzerland

**Keywords:** Pre-diabetes, Diabetes, Non-communicable disease, Blood glucose, Awareness, Treatment, Control, Kenya

## Abstract

**Background:**

Diabetes Mellitus is one of the four major non-communicable diseases causing about 4 million deaths in 2017. By 2040, low income countries are projected to experience 92% increase in mortality due to diabetes. Undiagnosed diabetes poses a public health concern with costly public health implications especially in Africa. It is therefore crucial to examine the burden and risk factors for diabetes at national level to inform policy and national programs.

**Methods:**

Data from the 2015 Kenya national STEPs survey of adults aged 18–69 years were used. Pre-diabetes was defined as impaired fasting blood glucose level (6.1 mmol/l to < 7 mmol/l) while diabetes was defined as impaired fasting blood glucose level ≥ 7 mmol/l. Descriptive statistics were used to determine the prevalence of pre-diabetes and diabetes and logistic regression was used to identify associated factors.

**Results:**

Complete data for 4069 respondents (51% females), with 46% aged 18–29 and 61% in rural areas were analyzed. The age-standardized prevalence for pre-diabetes and diabetes were 3.1% (95% CI: 2.2, 4.0) and 2.4% (1.8, 3.0) respectively. Only 43.7% were aware of their glycemic condition, one in five of those who had diabetes had received treatment, and only 7% of those diagnosed with diabetes had their blood glucose under control. Primary education ((both incomplete (0.21, 95%CI 0.10–0.47) and complete (0.40, 95%CI 0.23–0.71)) were associated with lower odds of pre-diabetes. Older age (60–69 years, AOR; 5.6, 95%CI 2.1–15.1) and raised blood pressure (2.8, 95% CI 1.5–5.0) were associated diabetes while overweight/obesity among women was associated with diabetes.

**Conclusion:**

The overall diabetes prevalence in Kenya is consistent with what has been reported in other sub-Saharan African countries. Of concern is the higher prevalence of pre-diabetes and undiagnosed diabetes that can progress to complications in the absence of interventions and the low diabetes awareness and control. This is the first nationally representative study to identify important groups at risk of pre-diabetes and diabetes that can be targeted for screening, health promotion and treatment.

## Background

Diabetes Mellitus is one of the four major non-communicable diseases (NCDs) comprising - cardiovascular diseases, cancers and chronic respiratory diseases jointly contributing to 63% of NCD deaths worldwide [1, 2]. From 1980 to 2014 the number of people living with diabetes globally increased from 108 million to 442 million [3, 4]. In 2017, the estimated figure had risen to 425 million people around the world and the majority were in low and middle income countries [5] with direct annual cost on the world estimated at US$825 billion [2]. Mortality from diabetes occurred every 8 seconds in 2017 estimated at 4 million among 20–79 year olds [5]. If the current diabetes trends continue unchanged, both the number of people living with diabetes and the deaths from diabetes are expected to increase. Low income countries are expected to experience the highest increase in diabetes prevalence (92%) followed by lower-middle income countries (57%), upper- middle income countries (46%) and higher income countries (25%) [3, 4, 6].

Undiagnosed cases of diabetes are a public health concern with costly public health implications. Globally, undiagnosed diabetes are common. Worldwide estimates for undiagnosed diabetes was 50% among people 20–79 years; the proportion of undiagnosed diabetes in Africa (69%) is almost double that of high-income countries (37%) [5]. This contributes to the high morbidity and mortality burden, which occurs at a younger age in Africa. Undiagnosed individuals with diabetes are likely to experience complications even before a diagnosis is made [5]. This can have additional cost implications for households [7] and on already overburdened health systems [8], thus a need to increase screening efforts worldwide to prevent the progression to diabetes.

Pre-diabetes, defined as glycemic levels that are higher than normal, but lower than diabetes thresholds (fasting glucose > 6·0 mmol/L and < 7·0 mmol/L) is considered an important risk factor for diabetes and its associated complications such as nephropathy, diabetic retinopathy, and increased risk of macrovascular disease [4, 9–11]. Thus, understanding pre-diabetes is important for future diabetes projections. Some national studies in the US and China estimate adult pre-diabetes prevalence of 36.2% and 50.1%, respectively [12, 13]. One study projects that by 2030, 470 million people will have pre-diabetes globally [14]. Studies have suggested that progression to diabetes is occurring among those diagnosed with pre-diabetes. Tabak et al. suggested that the risk of people with pre-diabetes developing diabetes is 5–10% per year [15]. Larson et al. in a study among postmenopausal women showed that 25% of subjects with impaired fasting glucose (IFG) or impaired glucose tolerance (IGT) test progressed to type 2 diabetes (T2DM) in 5 years [16]. However, there is also evidence indicating that managing pre-diabetes with lifestyle modifications such as physical activity [17] and healthy diets [18, 19] can prevent or delay the progression of pre-diabetes to diabetes while another study reported that behavioral modifications alone reduced the risk of diabetes by 40–70% [15]. It is thus important to identify people in the pre-diabetic state for timely preventive interventions.

Previous population based studies in both rural and urban Kenya found a diabetes prevalence of 3.5–5%, with higher proportions among those in the urban areas [20–23]. These studies are not nationally representative because they lacked national geographic coverage. In 2015, the international Diabetes Federation (IDF) estimated the diabetes prevalence for Kenya to be 2.2% [24]. However the IDF estimates are based on a combination of several data sources including health facility data, small population studies and modelling that may not provide robust estimates. There is need for empirical data at population level to accurately determine the true burden of diabetes in Kenya. Therefore, this study seeks to determine the prevalence, awareness, treatment and control of diabetes and its determinants in a nationally representative sample in order to inform national diabetes programs and resource allocation.

## Methods

### Study site and population

Data for this study were obtained from the 2015 Kenya STEPs survey. This was the first national household survey on NCD risk factors. Data was collected in all 47 counties in Kenya between April and June 2015. The aim of the overall study was to provide estimates for indicators on NCD risk factors for persons aged 18–69 years.

### Sample size and sampling

A multistage stratified sampling method was used to allow national estimates by sex (male and female) and residence (urban and rural). The survey used the fifth National Sample Surveys and Evaluation Programme (NASSEP V) master sample frame that was developed by the Kenya National Bureau of Statistics (KNBS). The frame was developed using the Enumeration Areas (EAs) generated from the 2009 Kenya Population and Housing Census to form 5360 clusters split into four equal sub-samples. A total of 6000 households were sampled targeting one individual randomly selected from the eligible household members. A total 4754 households gave consent to participate in the study. To produce unbiased estimates, sampling weights were calculated as the inverse or reciprocal of all the selection probabilities at all the stages mentioned above. The weights were derived from the processes involved in the creation of sampling frame (NASSEP V) and selection of individuals in the study. Further, the weights were adjusted to cover individual non-responses. Post stratification adjustments were done to align with the population projections according to age-sex categories.

### Data collection

Data were collected by trained personnel using a structured questionnaire adapted from the WHO STEPwise approach to chronic disease risk factor surveillance (STEPS) tool [25] with modifications to suit the Kenyan context. Data was collected in 2 days. On the first day, the questionnaire was administered and anthropometric measurements were also collected. Participants were asked on day one to fast overnight and fasting blood measurements were collected on day two. The questionnaire elicited information on demographic characteristics and health behaviors. The trained field personnel took anthropometric measurements (blood pressure, heart rate, height, weight, waist and hip circumference), and biochemical measurements (fasting blood glucose, and lipid profile (total cholesterol and HDL cholesterol only)).

Data were recorded on Personal Digital Assistants (PDAs) loaded with eSTEPS software. Further details on the data collection and quality assurance procedures are described elsewhere [26].

### Measurements and definitions

Diabetes measurements and definitions were based on established guidelines by the WHO [10]. Fasting blood glucose levels were measured using a point of care instrument (CardiocheckPA analyzer®) from PTS Diagnostics. Pre-diabetes was defined as impaired fasting blood glucose (IFG) of 6.1 mmol/l to less than 7 mmol/l while diabetes was defined as fasting blood glucose of 7 mmol/l or more or a self-report of previous diagnosis of diabetes by a health care professional or currently receiving treatment for diabetes. Awareness was defined as prior diagnosis of diabetes by a healthcare professional among all participants. Treatment was defined as receiving pharmacologic treatment to lower blood glucose in the previous 2 weeks among all diabetic participants. Control was defined as having a fasting blood glucose of < 7 mmol/l while on pharmacologic treatment among all diabetic participants.

Fasting lipid levels were also were measured using a point of care instrument (CardiocheckPA analyzer®) from PTS Diagnostics. Abnormal lipid values were defined using National Cholesterol Education Program (NCEP) guidelines [27]. High cholesterol was defined as total cholesterol ≥5.0 mmol/L or are currently on medication for raised cholesterol. Low HDL-cholesterol (HDL-C) was defined as HDL-C < 1 mmol/l for men and < 1.3 mmol/l for women.

Blood pressure was measured using a validated blood pressure machine (OMRON M2 device). Three readings were taken for systolic blood pressure (SBP) and diastolic blood pressure (DBP) in accordance with the WHO hypertension guidelines [28]. The averages of the last two readings were then recorded. Hypertension (High/raised blood pressure) was defined as a systolic blood pressure (SBP) ≥ 140 mmHg, or diastolic blood pressure (DBP) ≥ 90 mmHg, or previous diagnosis of hypertension or being on antihypertensive therapy [28].

Standing height without shoes was measured to the nearest 0.1 cm using a portable height measuring equipment (SECA 877). Weight was also measured to the nearest 0.1 kg with participants wearing light clothing and without shoes using a pre-calibrated digital weighing scale (SECA 877).

Body mass index (BMI) was calculated as weight divided by height squared (kg/m^2^). Overweight was defined as a BMI ≥ 25–29.9 kg/m^2^ and obesity was defined as BMI ≥ 30 kg/m^2^ using the WHO BMI cut-off points [29].

Waist circumference was measured using a constant tension tape across the umbilicus level. Central obesity was defined as waist circumference ≥ 94 cm for men and ≥ 80 cm for women [29, 30].

Physical activity was self-reported using the WHO Global Physical Activity Questionnaire [31] which was added to the main study questionnaire. Insufficient physical activity in this study was defined as self-reports of less than 150 min of moderate intensive activity or less than 75 min of vigorous intensive physical activity per week, including walking, running and cycling.

Harmful use of alcohol was defined as consumption of more than 1 standard drink (which is the amount of alcohol found in a small beer, one glass of wine, or one tot of spirits) per day for females and more than 2 standard drinks for males [32, 33].

Tobacco use was defined as self-reported current use of smoked tobacco or smokeless tobacco products.

High sugar intake was defined as self-reports of far too much or too much consumption of sugar in a day. Bad fat intake was defined as self-reported use of saturated fats e.g. lard, margarine, butter and vegetable fat for cooking. High salt consumption was defined as self-report of far too much or too much consumption of actual salt and in processed foods, adding salt when cooking and/or to cooked food. Insufficient fruit and vegetable intake was defined as self-reported consumption of less than 5 servings/day of fruit and vegetables.

Socio-demographic variables considered for this study included age recoded into four categories (18–29, 30–44, 45–59, and 60–69). At the time of data collection, the current education system in Kenya was the 8–4-4 model which had 8 years of primary education, 4 years of secondary education and 4 years of university. In this study education was categorized into four groups [i) no schooling, ii) primary incomplete – those who did not complete the 8 years of primary school, iii) primary complete – those who completed 8 years of primary school and iv) secondary level and more – those with either secondary education and higher)]. Occupation was categorized in three categories [i) employed – those on salary employment, ii) self-employed – those with businesses including small businesses and iii) unemployed – those not working at all]. Marital status was categorized into two categories [i) *in union* – includes those married and those not married but living together and ii) *not in union* – those not married].

Socio-economic status was measured using a household asset and amenities index that assessed household ownership of various assets and amenities commonly used in Demographic and Health Surveys in low and middle income countries [34]. Standardized weight scores were generated using principal components analysis and ranked to generate wealth quintiles which were recoded into five quintiles from the lowest representing poorest households to highest quintile representing the wealthiest households.

### Data analysis

The analytical dataset used in this study excluded 15 participants with inconsistent age data. We computed proportions to assess the prevalence, awareness, treatment and control of pre-diabetes and diabetes. The direct method was used to estimate the age standardized prevalence’s’ for pre-diabetes and diabetes using the 2009 Census data to adjust the proportions. We further employed logistic regression models to examine the demographic, behavioral, and body composition factors associated with the prevalence of pre-diabetes and diabetes. All the known CVD risk factors in the dataset were added to the models. Demographic variables included in the model were age, sex, education, marital status, place of residence, and household socio-economic status.

All the analysis were done separately for males and females in addition to an overall model for both. Age-standardized estimates were computed using the 2009 Census data to adjust the proportions. *P*-values less than 0.05 were considered to be statistically significant. All data analyses were weighted using individual weights to reflect national representativeness. All analyses were done using STATA version 14 (Stata Corporation, College Station, TX).

### Ethical approval

The study protocol was reviewed and approved by the Kenya Medical Research Institute’s Ethics Review Committee (SSC No. 2607). All study participants were informed about the study aims including both potential benefits and risks associated with participation. Verbal consent was sought from the household head. All eligible participants gave informed written consent before interview and examination. During the survey, participants who were noted to have abnormalities in their laboratory tests and blood pressure measurements were referred for further care to the nearest health facility or facility of their choice with a referral form. Patient identifiers were delinked from the analytical datasets.

## Results

### Demographic and lifestyle characteristics

A total of 4500 eligible individuals were successfully interviewed among the sampled 6000 individuals yielding a response rate of 75%. The study enrolled 4164 while 336 individuals were excluded (14 participant were below 18 years, 1 participant was above 69 years, 321 did not participate in the third step (biochemical measurements). Complete data for fasting glucose measurement were available for 4069 (98%) eligible participants (95 had missing fasting blood glucose values).

Table 1 summarizes socio-demographic characteristics of the study participants. About half of the respondents were aged 18–29 years (46%), 51% were females. A significantly higher proportion of women, compared with men, had no schooling (18.2% vs. 6.5%), lived in rural areas (64.4% vs. 57.4%), were unemployed (53.8% vs. 25.6%), were in marital union (69% vs. 63.2%) and were from the poorest wealth quintile (21.8% vs. 16.5%).

**Table 1:** Socio-demographic characteristics by gender

Indicator	Total %	Female %	Male %	*p*-value
Age groups (Years)				
18–29	46.1	47.2	44.9	0.736
30–44	32.7	32.0	33.4	
45–59	15.9	15.6	16.3	
60–69	5.3	5.3	5.4	
Education level				
No Schooling	12.5	18.2	6.5	< *0.001*
Primary incomplete	23.3	23.5	23.0	
Primary complete	32.4	33.9	30.7	
Secondary+	31.9	24.4	39.7	
Residence				
Rural	61.0	64.4	57.4	*0.021*
Urban	39.0	35.6	42.6	
Occupation				
Employed	21.0	11.6	30.9	< *0.001*
Self-employed	38.9	34.5	43.5	
Unemployed	40.0	53.8	25.6	
Marital status				
Not in union	33.9	31.0	36.8	*0.033*
In union^a^	66.1	69.0	63.2	
Wealth status				
Poorest	19.2	21.8	16.5	*0.015*
Second	21.0	21.8	20.2	
Middle	17.7	18.6	16.9	
Fourth	18.4	16.4	20.5	
Richest	23.7	21.5	25.9	
Total (N)	4069	2462	1607	

^a^includes not married but living together

*italicized* *p*-values are significant

Table 2 summarizes clinical risk factors of the study participants. Women compared with men were significantly more likely to have abdominal obesity (50.2% vs. 12.1%), BMI ≥25 kg/m^2^ (39.1% vs. 17.4%), low HDL-cholesterol (59.9% vs. 45.9%) and high total cholesterol levels (9.8% vs. 4.6%). Men, compared with women, were significantly more likely to demonstrate risky behaviors such as current tobacco use (22.8% vs. 3.9%), harmful alcohol use (26.3% vs. 4.8%) and bad fat intake (43.7% vs. 35.3%).

**Table 2:** Risk factors for diabetes by gender

Indicator	Both		Female		Male		*p*-value
	%	n	%	n	%	n	
Insufficient Physical activity^a^	80.1	3262	81.2	1999	79.1	1263	0.266
Waist circumference obesity ^b^	31.6	1483	50.2	1287	12.1	196	<* 0.001*
High sugar intake ^c^	15.0	584	13.5	353	16.5	231	0.105
BMI ≥25 kg/m2	28.1	1224	39.1	912	17.4	312	< *0.001*
Low HDL cholesterol ^e^	53.1	2207	59.9	1201	45.9	1006	< *0.001*
High blood pressure^d^	24.8	1147	23.6	693	26.0	454	*0.178*
Current tobacco use ^f^	13.2	485	3.9	104	22.8	381	<* 0.001*
Harmful use of alcohol ^g^	15.3	469	4.8	82	26.3	387	<* 0.001*
Bad fat intake ^h^	39.4	1686	35.3	939	43.7	747	*0.002*
High salt consumption ^i^	89.6	3567	89.1	2151	90.1	1416	0.437
Diabetes ^j^	2.5	135	2.9	42	2.1	93	0.163
Prediabetes ^k^	3.1	142	3.2	55	2.9	87	0.667
High total cholesterol ^l^	7.3	480	9.8	341	4.6	139	*0.001*
Mean total cholesterol [95% CI]	5.1	[3.0;7.2]	4.0	[2.9;5.0]	6.2	[2.3;10.2]	
Mean HDL cholesterol [95% CI]	1.19	[1.16;1.22]	1.12	[1.08;1.17]	1.26	[1.21;1.30]	
Mean glucose [95% CI]	4.6	[4.5;4.7]	4.5	[4.4;4.6]	4.7	[4.6;4.8]	
Mean systolic blood pressure [95% CI]	124.2	[123.1;125.2]	126.9	[125.5;128.3]	121.6	[120.3;122.9]	
Mean diastolic pressure [95% CI]	80.4	[79.7;81.1]	80.2	[79.2;81.3]	80.6	[79.8;81.3]	
Mean age [95% CI]	34.3	[33.4:35.3]	34.6	[33.4;35.9]	34.0	[33.1;35.0]	
Total (N)	4069	4069	2462	2462	1607	1607	

^a^Insufficient physical activity in this study was defined as self-reports of less than 150 min of moderate intensive activity or less than 75 min of vigorous intensive physical activity per week, including walking and cycling, ^b^ Central obesity was defined as waist circumference ≥ 94 cm for men and ≥ 80 cm for women, ^c^ High sugar intake was defined as self-reports of far too much or too much consumption of sugar in a day, ^d^ High blood pressure was defined as a systolic blood pressure (SBP) ≥ 140 mmHg, or diastolic blood pressure (DBP) ≥ 90 mmHg, or previous diagnosis of hypertension or being on antihypertensive therapy, ^e^ Low HDL-cholesterol (HDL-C) was defined as HDL-C < 1 mmol/l for men and < 1.3 mmol/l for women, ^f^ Tobacco use was defined as self-reported current use of smoked tobacco or smokeless tobacco products, ^g^ Harmful use of alcohol was defined as consumption of more than 1 standard drink (which is the amount of alcohol you find in a small beer, one glass of wine, or one tot of spirits) per day for females and more than 2 standard drinks for males, ^h^ Bad fat intake was defined as self-reported use of saturated fats e.g. lard, margarine, butter and vegetable fat for cooking, ^I^ High salt consumption was defined as self-report of far too much or too much consumption of actual salt y and in processed, adding salt when cooking and/or to cooked food, ^j^ diabetes was defined as fasting blood glucose of 7 mmol/l or more or a self-report of previous diagnosis of diabetes by a health care professional or currently receiving treatment for diabetes, ^k^ Pre-diabetes was defined as impaired fasting blood glucose (IFG) of 6.1 mmol/l to less than 7 mmol/l, ^l^ High cholesterol was defined as total cholesterol ≥5.0 mmol/L or are currently on medication for raised cholesterol, Missing data: Physical inactivity (52), sugar intake (1), Hypertension (16), HDL (1), BMI (155), alcohol intake (5), fat intake (3), total cholesterol (1), *P*-values derived from chi-square test

*italicized* *p*-values are significant

Mean age, glucose, systolic blood pressure, diastolic blood pressure and mean serum levels of total cholesterol, and HDL-C by sex are also presented in Table 2. The mean age in years for the total sample was 34.3 (men, 34.6 and women, 34.0). Compared to women, men had higher mean total cholesterol (6.2 vs. 4.0), HDL-C (1.26 vs. 1.12), mean glucose (4.7 vs. 4.5) and mean diastolic pressure (80.6 vs. 80.2). Compared to men, women had higher mean systolic blood pressure (126.9 vs. 121.6) and age (34.4 vs. 34.0).

### Prevalence of pre-diabetes and diabetes

The overall age adjusted prevalence for pre-diabetes was 3.1% (95% CI: 2.2, 4.0) and prevalence of diabetes was 2.4% (1.8, 3.0). The age standardized prevalence of pre-diabetes was higher among respondents from urban areas (3.5%) than rural areas (2.7%), among female (3.3%) compared to male (2.8%) and those in the richest wealth quintile (4.9%) compared to poorest quintile (3.3%) though these were not statistically significant. Similarly, the age adjusted prevalence of diabetes was highest among urban (3.4%) compared to rural (1.9%) residents and highest among the respondents in the 4th richest wealth quintile and 5th richest wealth quintile (5.2%) compared to respondents in the poorest wealth quintile (1.6%). These differences were statistically significant (Table 3). The proportion of undiagnosed diabetes among the study participants was 52.8% (50.7% among females and 55.9% among males) [Table not shown].

**Table 3:** Age adjusted prevalence of pre-diabetes and diabetes by residence, wealth status and region, STEPS survey

Indicator	Diabetes (% [95%CI])	Pre-diabetes (% [95%CI])	Diabetes awareness (% [95%CI])	Diabetes treatment (% [95%CI])	Diabetes control (% [95%CI])
National	2.4 [01.8:03.0]	03.1 [02.2:04.0]	43.7 [29.1:59.5]	21.3 [12.0:35.1]	07.0 [03.6:13.2]
Residence					
Rural	1.9 [01.3:02.5]	02.7 [01.8:03.7]	39.5 [25.2:55.7]	14.9 [08.6:24.6]	07.7 [03.3:17.1]
Urban	3.4 [02.1:04.7]	03.5 [01.6:05.3]	48.6 [22.4:75.6]	28.7 [11.1:56.4]	06.2 [02.2:16.2]
Wealth status					
1 - Poorest	1.6 [00.7:02.5]	03.3 [01.1:05.5]	17.8 [04.0:52.9]	00.0 [00.0:00.0]	00.0 [00.0:00.0]
2	1.5 [00.7:02.2]	02.3 [01.1:03.4]	53.0 [27.1:77.3]	23.1 [08.7:48.7]	13.2 [03.8:37.1]
3	2.0 [01.1:02.9]	03.0 [01.4:04.6]	32.5 [16.2:54.5]	28.4 [13.3:50.7]	17.6 [06.8:38.5]
4	3.0 [01.8:04.3]	01.6 [00.7:02.6]	49.5 [23.7:75.5]	33.2 [10.3:68.2]	02.5 [00.6:09.8]
5 - Richest	5.2 [02.5:07.9]	04.9 [01.4:08.4]	52.9 [16.5:86.5]	16.0 [04.5:43.4]	04.5 [00.9:19.2]
Sex					
Female	2.8 [02.0:03.6]	03.3 [02.2:04.4]	44.9 [28.6:62.4]	33.7 [19.8:51.3]	10.5 [05.4:19.6]
Male	2.0 [01.1:02.9]	02.8 [01.6:04.1]	42.0 [21.8:65.2]	03.5 [01.1:10.6]	01.9 [00.3:09.7]
Age (years)					
18–29	0.6 [00.3;01.4]	02.1 [01.1;03.8]	27.8 [05.8:70.6]	08.2 [01.6:32.8]	05.3 [00.6:32.7]
30–44	2.3 [01.5;03.5]	02.9 [02.0;04.2]	21.7 [09.2:43.0]	16.1 [06.7:34.1]	09.0 [02.9:24.5]
45–59	7.1 [04.8;10.3]	05.3 [03.5;07.8]	54.9 [32.0:75.9]	28.5 [12.2:53.2]	06.0 [02.2:15.3]
60–69	6.0 [03.7;09.5]	06.4 [03.9;10.2]	70.1 [45.7:86.8]	20.1 [08.0:42.2]	07.5 [02.4:21.2]
Total (N)	4069	3934	135	135	135

NB: Awareness, treatment and control estimates are not age adjusted. Awareness was defined as prior diagnosis of diabetes by a healthcare professional among diabetic participants. Treatment was defined as receiving pharmacologic treatment to lower blood glucose in the previous 2 weeks among diabetic participants. Control was defined as having a fasting blood glucose of < 7 mmol/l while on pharmacologic treatment among diabetic participants

### Prevalence of awareness, treatment and control of diabetes

Among participants diagnosed with raised fasting blood glucose or currently on medication for diabetes, 43.7% were aware of their condition (Table 3). Awareness was higher among respondents from urban areas (48.6%) compared to rural areas (39.5%), those in the highest wealth quintile (52.9%) compared to lowest wealth quintile (17.8%), among females (44.9%) compared to males (42.0%) and among the oldest (60–69) age group (70.1%) compared to the youngest (18–29) age group (17.8%) though these differences were not statistically significant. One in five of the respondents diagnosed with diabetes had received treatment for the condition, with more respondents from urban areas (28.7%), females (33.7%) and older age groups (28.5%, 45–59, 20.1%, 60–69) compared to respondent from rural areas (14.9%), males (3.5%) and younger age group (8.2%, 18–29). However, all these were not statistically different. All individuals from the poorest household were not on treatment compared to the households in the other wealth quintile and this difference was statistically significant. Among those who were aware of their diagnosis, only 41% were on treatment [Table not shown]. Less than a tenth (7%) of the respondents diagnosed with diabetes had their blood glucose level controlled. Among those on treatment only 33% had achieved glycemic control [Table not shown]. Similar to treatment levels, individuals with diabetes from the poorest households were not controlled for diabetes and this was statistically significant (0.0, 95% CI 0.0–0.0).

### Predictors of pre-diabetes and diabetes

#### Factors associated with pre-diabetes

Overall, education level was the only factor associated with pre-diabetes; individuals with both incomplete primary (0.22, 95% CI 0.10–0.47) and complete primary (0.40, 95% CI 0.22–0.70) education had lower odds of having pre-diabetes compared to individuals having no formal education [table not shown]. Women with high total cholesterol had higher odds for pre-diabetes compared with those with normal total cholesterol levels ([adjusted odds ratio] AOR 1.98).

#### Factors associated with diabetes

Individual’s age and raised blood pressure were found to be significantly associated with diabetes (Table 4). The odds for diabetes increased with older age and was highest in 45–59 year old participants compared with 18–29 year olds (AOR 6.59). Individuals with raised blood pressure had higher odds for diabetes compared with those with normal blood pressure (AOR 2.8). Females with BMI levels ≥25 kg/m^2^ (overweight/obese) had higher odds for diabetes compared to those with normal BMI levels (AOR 3.2).

**Table 4:** Determinants of diabetes, overall and by gender

Indicator	Both		Males		Females	
	AOR [95%CI]	*P*-value*	AOR [95%CI]	*P*-value*	AOR [95%CI]	*P*-value*
Age groups (Ref: [18–29])	1.00		1.00		1.00	
30–44	2.55 [0.89;7.28]	*< 0.001*	1.32 [0.13;13.47]	*0.001*	*3.92 [1.19;12.88]*	*0.035*
45–59	*6.59 [2.55;17.07]*		7.98 [0.85;74.75]		*5.23 [1.74;15.74]*	
60–69	*5.64 [2.11;15.13]*		7.63 [0.99;58.78]		*4.01 [1.15;13.96]*	
Sex (Ref: Females)	1.00					
Males	0.76 [0.39;1.49]	0.420				
Hypertensive (Ref: Normal BP)	1.00		1.00		1.00	
Raised BP	*2.77 [1.53;5.03]*	*0.001*	2.29 [1.00;5.26]	0.051	*3.32 [1.70;6.51]*	*0.001*
Waist circumference (Ref: Normal)	1.00		1.00		1.00	
Obese	2.02 [0.94;4.37]	0.073	3.40 [0.71;16.39]	0.126	1.39 [0.72;2.68]	0.324
Body mass index (Ref: Normal)	1.00		1.00		1.00	
Obese/Overweight	1.64 [0.73;3.70]	0.228	0.79 [0.15;4.04]	0.774	*3.16 [1.70;5.88]*	*< 0.001*
Insufficient physical activity (Ref: Sufficient)	1.00		1.00		1.00	
Insufficient	2.02 [0.86;4.71]	0.104	2.81 [0.74;10.63]	0.127	1.40 [0.60;3.24]	0.436
Sugar intake (Ref: Normal)	1.00		1.00		1.00	
High	1.44 [0.77;2.70]	0.249	1.22 [0.34;4.32]	0.762	1.82 [0.81;4.11]	0.148
Harmful use of alcohol (Ref: No)	1.00		1.00		1.00	
Yes	1.88 [0.84;4.21]	0.126	2.14 [0.79;5.83]	0.134	1.62 [0.30;8.86]	0.577
Fat intake (Ref: Good)	1.00		1.00		1.00	
Bad	0.78 [0.45;1.34]	0.361	0.62 [0.24;1.62]	0.328	0.84 [0.49;1.44]	0.529
HDL cholesterol (Ref: Normal)	1.00		1.00		1.00	
Low	0.72 [0.40;1.29]	0.265	0.87 [0.39;1.96]	0.740	0.66 [0.37;1.20]	0.173
Total cholesterol (Ref: Normal)	1.00		1.00		1.00	
High	1.47 [0.83;2.60]	0.182	1.55 [0.36;6.60]	0.555	1.89 [0.88;4.03]	0.101
Total (N)	3877		1577		2300	

Place of residence, marital status, education level and wealth status were not significant hence results not presented here

* Italicized *estimates are significant

*this is the overall *p*-value for the association between between the dependent and independent variables

## Discussion

In this study we report the findings of the first nationally representative estimates for the burden of diabetes and pre-diabetes, and the awareness, treatment and control of diabetes among Kenyan adults. We also examined the variables associated with pre-diabetes and diabetes. The results show a fairly low diabetes prevalence (2.4%) but with a substantial proportion (52.8%) of people with undiagnosed diabetes that are at risk of developing complications. This provides a baseline assessment against which progress in prevention and control efforts can be measured.

The estimated diabetes prevalence of 2.4% found in this study is similar to the national prevalence of 2.2% reported by IDF for Kenya in 2015 [24] but lower than estimates (4%) by Christensen et al. in 2009 [20] and WHO in 2016 [35]. The possible differences in Christensen’s estimate and the current study is that the current study was a nationally representative sample while the Christensen’s sample was from a few selected ethnic groupings in Kenya. Similarly, the WHO estimate is also based on multiple data sources and several assumptions are used to model the population estimate. Therefore, the results of the current study are more robust owing to nationally representative sample and a more reliable method of data collection. The diabetes prevalence (3.4%) in the urban population was slightly lower than the estimate (4.8–5.3%) reported in the slums of Nairobi [21, 22]. As the largest city, it is likely that Nairobi may be experiencing a faster epidemiological transition than other urban communities in Kenya.

Kenya’s diabetes prevalence of 2.4% is much higher than that of the neighboring country, Uganda with a prevalence of 1.4% from the recent STEPs survey [36]. The pre-diabetes prevalence of the current study was 3.1%. Malawi and Ghana reported diabetes cut off points that include the current study’s pre-diabetic sample and found a prevalence similar to the combined diabetes and pre-diabetes prevalence in the current study [37, 38]. Similar to studies conducted in Uganda [36] and South Africa [39] this study reported significantly higher diabetes prevalence in urban areas. This can be attributed to a faster epidemiological transition in urban areas associated with urbanization [40].

As observed in many settings [6, 41–43], pre-diabetes prevalence is higher than diabetes prevalence in our study. Available literature shows that persons with diabetes pass through a pre-diabetic phase during which if no prevention measures are instituted, they become diabetic [44, 45]. Pre-diabetes is a known risk factor for type 2 diabetes [46–48]. It is therefore critical to identify groups at risk of pre-diabetes for lifestyle changes to prevent progression to diabetes. In our study we identified two groups; both men and women with no formal education and women with high total cholesterol levels to be at risk of pre-diabetes. Any form of formal education was associated with lower odds of pre-diabetes as shown by studies in Sweden, China and other European countries [49–51]. Education improves access to relevant prevention messages and comprehension of such information thus influences health behaviors [52].

Diabetes on the other hand was associated with older age, raised blood pressure, and obesity among women as established in literature on metabolic syndrome [53]. The strong associations between advancing age and diabetes [20, 36], obesity and diabetes are well established [22]. A study by Wong-McClure found higher BMI and low education to be significantly associated with diabetes which is consistent with what the current study found [54].

The most worrying finding is the low level of awareness of diabetes among adults in Kenya with less than half the participants diagnosed with diabetes reporting that they were aware of their status. However, this is comparable to awareness levels found in most SSA countries suggesting poor access to screening services in these countries [23, 36, 55, 56]. While Kenya’s awareness level of 43.7% is low, it is lower than Uganda’s awareness level of 51.1% [36] but higher than Tanzania’s awareness level of 35.6% [57]. We found higher levels of awareness among urban residents, those from wealthier households but these were statistically insignificant. The high level of unawareness is worrying because of the impending diabetes complications that can be prevented if timely diagnosis is available for prompt management.

Our results also show that diabetes treatment levels are low. Only 21.3% of the patients with diabetes reported that they were on treatment while only 41% among those who were aware of their diagnosis were on treatment. The low levels of awareness of having diabetes is likely to contribute to low levels of treatment. The high treatment costs associated with diabetes care is also likely to contribute to the low treatment levels among those who are aware of their condition. A recent Lancet commission report on diabetes in SSA concluded that costs associated with diabetes treatment are high for patients in many countries since they mainly (> 50%) pay out-of-pocket [58]. The household expenditure and utilization survey in Kenya revealed only one in every five people seek and access health care and this was mainly due to the prohibitive treatment costs [59]. Women had a higher uptake of diabetes treatment than men as cited in a recent survey that revealed more frequent healthcare visits among women than men [59]. In addition to the frequent healthcare visits the higher treatment levels in women may be due to the higher awareness levels among women as well.

Glycemic control is important in prevention of complications and premature death. A study in the referral Kenyatta National Hospital showed a high case fatality in patients with diabetic ketoacidosis due to uncontrolled levels of glycaemia [60]. Our results show that glycemic control was only achieved by 7% of patients with diabetes and by 33% among those on treatment. These low control levels could be a result of the many factors including inaccessibility to tests and medications, low adherence to treatment regimens by the patients as well as drug stock outs increasing the number of patients missing prescribed medications. A meta-summary of diabetes in SSA found that inaccessibility to and the high cost of diabetes treatment was associated with poor glycemic control [23]. Similar to treatment levels, none of the patients with diabetes in the poorest households achieved glycemic control and this is also likely due to not accessing treatment. Diabetes control was significantly high among women and this could be due to the higher proportion of women being on treatment.

### Study strengths and limitations

Strengths of the current study include the use of a large national sample that has provided a complete national picture of diabetes situation in Kenya. The cross-sectional nature of this study precludes any causal association. The reliance of self-reported physical activity, dietary data and social behaviors may lead to incorrect estimates. Another potential limitation in a population based study such as this is the uncertainty of achieving a fasting state by the clients which could easily lead to an over-estimate of diabetes prevalence. However, during data collection, fasting blood samples were collected 1 day after the interview at a central hub to allow participants follow fasting advice given on the day of interview.

## Conclusions

This study provides the first national estimate for pre-diabetes and diabetes in Kenya and adds to the body of knowledge on diabetes and pre-diabetes in the country. The study results suggest a slightly higher prevalence for pre-diabetes than diabetes prevalence with a high proportion of patients with undiagnosed diabetes. This study thus provides an opportunity to inform interventions aimed at preventing the progression from pre-diabetes to diabetes and to its complications. In 2013 the WHO’s Global Action Plan 2013–2020 for NCDs called for a halt in the rise of diabetes and a 25% relative reduction in risk of premature mortality due to diabetes by 2020 [61]. It is time to heed the call to action on diabetes made a decade ago by African researchers [23]. Towards this, there is need in Kenya to support increased efforts towards promotion and prevention of diabetes among those at risk including; those older than 30 years, and those with raised blood pressure. Guidelines should be provided to healthcare workers to screen for these risk factors among these populations and sensitize them on appropriate care. Additionally, access to treatment among diabetic adults in Kenya should be prioritized in order to prevent the development of complications and premature deaths associated with uncontrolled diabetes.

## Abbreviation

CVD: Cardiovascular disease
DBP: Diastolic blood pressure
IDF: International Diabetes Federation
IFG: Impaired Fasting Glucose
IGT: Impaired Glucose Tolerance
KEMRI: Kenya Medical Research Institute
KNBS: Kenya National Bureau of Statistics
NASSEP: National Sample Surveys and Evaluation Programme
NCD: Non-communicable diseases
SBP: Systolic blood pressure
SSA: Sub-Saharan Africa
T2DM: Type 2 Diabetes Mellitus
